# Evolution of stridulatory mechanisms: vibroacoustic communication may be common in leaf-footed bugs and allies (Heteroptera: Coreoidea)

**DOI:** 10.1098/rsos.221348

**Published:** 2023-04-26

**Authors:** Michael Forthman, Chandler Downie, Christine W. Miller, Rebecca T. Kimball

**Affiliations:** ^1^ California State Collection of Arthropods, Plant Pest Diagnostics Branch, California Department of Food & Agriculture, 3294 Meadowview Road, Sacramento, CA 95832, USA; ^2^ Entomology & Nematology Department, University of Florida, 1881 Natural Area Drive, Gainesville, FL 32611, USA; ^3^ University of Florida, Gainesville, FL 32611, USA; ^4^ Department of Biology, University of Florida, 876 Newell Drive, Gainesville, FL 32611, USA

**Keywords:** Coreidae, Alydidae, stridulation, phylogeny, sequence capture, ultraconserved elements

## Abstract

Intra- and interspecific communication is crucial to fitness via its role in facilitating mating, territoriality and defence. Yet, the evolution of animal communication systems is puzzling—how do they originate and change over time? Studying stridulatory morphology provides a tractable opportunity to deduce the origin and diversification of a communication mechanism. Stridulation occurs when two sclerotized structures rub together to produce vibratory and acoustic (vibroacoustic) signals, such as a cricket ‘chirp’. We investigated the evolution of stridulatory mechanisms in the superfamily Coreoidea (Hemiptera: Heteroptera), a group of insects known for elaborate male fighting behaviours and enlarged hindlegs. We surveyed a large sampling of taxa and used a phylogenomic dataset to investigate the evolution of stridulatory mechanisms. We identified four mechanisms, with at least five evolutionary gains. One mechanism, occurring only in male Harmostini (Rhopalidae), is described for the first time. Some stridulatory mechanisms appear to be non-homoplastic apomorphies within Rhopalidae, while others are homoplastic or potentially homoplastic within Coreidae and Alydidae, respectively. We detected no losses of these mechanisms once evolved, suggesting they are adaptive. Our work sets the stage for further behavioural, evolutionary and ecological studies to better understand the context in which these traits evolve and change.

## 1. Introduction

For many insects, the ability to communicate information effectively between senders and receivers is critically important for survival and reproduction [1,2]. The forms and contexts of insect communication are diverse, such as pheromone trails of foraging ants (e.g. [3]), bioluminescent flashing for mate attraction in fireflies (e.g. [4,5]) and conspicuous warning colours in some weevils (e.g. [6]). Thus, auditory, chemical, visual and even tactile forms of communication may be involved with critical functions like mating, aggregating and defensive behaviours in insects. Why there is a diversity of communication systems across animals, as well as the factors that promote their origin and maintenance, has long been of interest to biologists and remains an active area of investigation (e.g. [7–9]).

The true bugs, or Heteroptera (Hemiptera), are no exception when it comes to expressing a diversity of communication signals. There are many examples of species exhibiting bright, contrasting colour patterns that have been shown or presumed to function as warning signals to would-be predators (e.g. [10–12]). Many species also have one or more scent glands, some of which might be used for, e.g. aggregation of conspecifics or to deter predators (e.g. [13,14]). Furthermore, some species can produce sounds and/or vibrations through the air or substrate (vibroacoustic communication), examples of which include percussion, tremulation or tymbal-like organs (e.g. [15,16]; reviewed in [17]). One other form of vibroacoustic communication that occurs in the Heteroptera is stridulation (e.g. [18–21]; also see [22]), which is also a common means of communication throughout invertebrates (e.g. [23–26]). It involves two sclerotized, ‘roughened’ structures; the plectrum is a movable structure that rubs against the stationary stridulitrum (or strigil) to produce both substrate (vibratory) and airborne (acoustic) signals with broadband frequency [27,28] (terminology reviewed by [20]; also see [22] for definitions followed in this study). In many Heteroptera, species lack a specialized auditory organ (i.e. tympanum) to receive airborne acoustic signals, and vibratory signals transmitted along substrates have been considered the primary form of intraspecific communication in such species (reviewed in [17]). However, stridulatory, substrate-borne vibratory signals or the airborne acoustic signals that accompany them might also convey information when conspecifics are in close proximity and/or in interspecific contexts [17]—intended or unintended, such as predator–prey interactions, as has been demonstrated in other groups of arthropods (e.g. [29]). Since the importance of the airborne component of stridulatory signals cannot be completely ruled out for these species, we use the term ‘vibroacoustic’ to describe the bimodal nature of their signalling.

The prevalence of stridulation within Heteroptera is unknown for many families, but, based on comparative morphological and taxonomic studies, the morphology of numerous species—particularly in the Nepomorpha, Gerromorpha, Reduviidae, Miridae, Pentatomoidea and Lygaeoidea—suggests it is common ([18–21,30,31]; also see [22]). Interestingly, the structures that are or are believed to be involved in stridulation vary widely among a broad range of taxa within the Heteroptera. Schuh & Weirauch [22] list 16 types of stridulatory mechanisms occurring in Heteroptera, which suggests a likely minimum of 16 independent gains of these structures and an importance of stridulation in many clades. However, phylogenetic comparative analyses investigating the evolution of stridulatory mechanisms within heteropteran clades are lacking.

**Figure 1.**
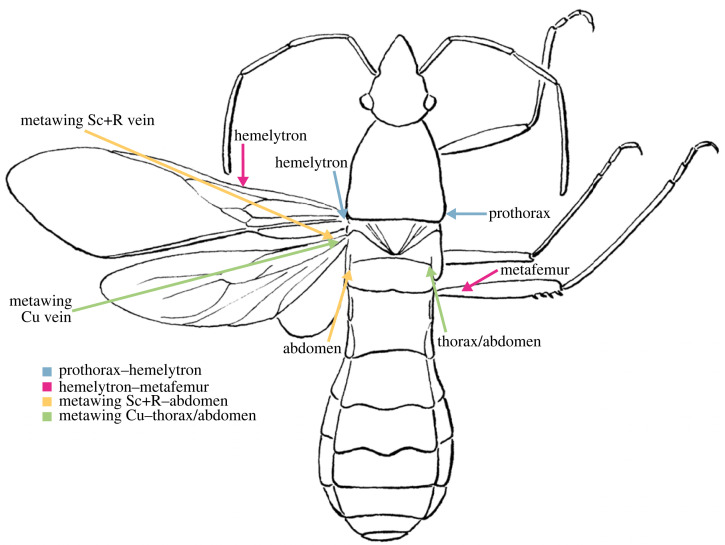
A generic diagram of an insect in Coreoidea (reproduced and modified from Schaefer & Pupedis [36] [their Fig. 17], Kansas Entomological Society), showing the relative positions of the structures involved in each stridulatory mechanism observed in this study. Cu, cubitus vein; Sc+R, subcosta + radius vein.

The leaf-footed bugs and allies (Coreoidea) are a globally distributed group of heteropterans, with about 3000 extant species classified in five families [32]. Coreoidea are morphologically diverse, include some agriculturally important species [33] and have been investigated in evolutionary ecology and behavioural studies primarily centered on sexual selection (e.g. [34,35]). Stridulatory signals and/or structures have been observed in both sexes for relatively few species within three families: Alydidae, Coreidae and Rhopalidae. So far, different stridulatory mechanisms have been documented among the families, with each having a specific mechanism (table 1, figure 1). In the Alydidae, the mechanism involves the ridged or ‘filelike’ anterior margin of the hemelytron and a patch of very fine tubercles on the basal region of the metafemur; the mechanism has been observed in five genera [36–38] (table 2). Within the Coreidae, acoustic signals were first observed in three species [39,41–43] (table 2), but it remained unclear as to what structures might be responsible for them for nearly a century. Štys [44] identified a stridulatory mechanism in three species (table 2); the plectrum consists of a long series of ridges on the posterolateral edge of the prothoracic foramen, and the stridulitrum involves the axillary sclerites and/or hypocostal lamina of the hemelytron. Interestingly, Štys [44] stated, ‘It is probable that…stridulation in Coreidae is not confined only to the 3 genera considered, because I have ascertained similar structures in several other genera. Some genera, however, certainly lack the stridulatory mechanism … ’, but he never mentions what other taxa were examined in the study. Since Štys' [44] publication, four additional species in the Coreidae have been reported to possess the same mechanism [40,45] (table 2). Miller [46] also reported a second stridulatory mechanism in the Coreidae, which involves the ventral surface of the clavus and the metathoracic wing (table 2). Lastly, *Jadera haematoloma* (Herrich-Schäffer, 1847) is the only species in the Rhopalidae known to stridulate, which involves the anterior-posterior movement of the basal abdominal tergites against the stridulitrum on the ventral surface of the metathoracic wing's subcosta + radius (Sc+R) vein [47].

**Table 1.** Stridulatory mechanisms observed in families of Coreoidea prior to this study. Sc+R, subcosta + radius vein.

family	plectrum	stridulitrum	stridulatory mechanism
Alydidae	basal posterior surface of metafemur	costal margin of hemelytron	hemelytron−metafemur
Coreidae	posterolateral edge of prothoracic foramen	axillary sclerites and/or hypocostal lamina of hemelytron	prothorax−hemelytron
Rhopalidae	dorsal lateral edge of abdominal tergite I^a^	ventral costal vein surface of metathoracic wing	metawing Sc+R−abdomen

^a^Plectrum requires anterior-posterior movement of abdominal tergites I and II to contact stridulitrum.

**Table 2.** Studies that have identified stridulatory mechanisms and/or sounds for taxa within the Coreoidea. Sc+R, subcosta + radius vein.

family	subfamily	tribe	taxon	stridulatory mechanism identified	sound observed
Alydidae	Alydinae		*Alydus* spp.	hemelytron–metafemur [34,35,37]	
Alydidae	Alydinae		*Burtinus* spp.	hemelytron–metafemur [34,37]	
Alydidae	Alydinae		*Euthetus* spp.	hemelytron–metafemur [34,37]	
Alydidae	Alydinae		*Megalotomus* spp.	hemelytron–metafemur [34,35,37]	
Alydidae	Alydinae		*Tollius* spp.	hemelytron–metafemur [34,35,37]	
Coreidae	Coreinae	Acanthocerini	*Euthochtha galeator* (Fabricius, 1803)	prothorax−hemelytron [39]	
Coreidae	Coreinae	Acanthocorini	*Rhyticoris terminalis* (Burmeister, 1835)	hemelytron–metawing [40]	
Coreidae	Coreinae	Coreini	*Centrocoris spiniger* (Fabricius, 1781)	prothorax−hemelytron [41]	[38]
Coreidae	Coreinae	Coreini	*Coreus marginatus* (Linnaeus, 1758)	prothorax−hemelytron [39]	
Coreidae	Coreinae	Coreini	*Spathocera laticornis* (Schilling, 1829)		[42]
Coreidae	Coreinae	Coreini	*Spathocera lobata* (Herrich-Schäffer, 1840)	prothorax−hemelytron [41]	
Coreidae	Coreinae	Gonocerini	*Cletomorpha raja* Distant, 1901	prothorax−hemelytron [39]	
Coreidae	Coreinae	Phyllomorphini	*Phyllomorpha laciniata* (Villiers, 1789)	prothorax−hemelytron [41]	[36,43]
Coreidae	Coreinae	Prionotylini	*Prionotylus brevicornis* (Mulsant & Rey, 1852)	prothorax−hemelytron [44]	
Rhopalidae	Serinethinae		*Jadera haematoloma* (Herrich-Schäffer, 1847)	metawing Sc+R−abdomen [45]	[45]

As is evident above, there have been few comparative morphological studies on stridulatory mechanisms within the Coreoidea (compared to studies with, e.g. Lygaeoidea, Nepomorpha and Gerromorpha), with all studies based on exceptionally limited taxon samples. While the presence of stridulatory mechanisms, in general, has likely evolved independently among the families of Coreoidea, it remains to be seen whether specific mechanisms have evolved once or multiple times within families. Indeed, based on relationships obtained in previous phylogenetic studies (e.g. [48–54]), some mechanisms may have evolved once and be synapomorphies that unite some clades (e.g. within the Alydidae), while others may have evolved multiple times and so are homoplastic in other clades (e.g. within the Coreidae). However, to understand the evolution of stridulatory mechanisms in the Coreoidea, a rigorous comparative analysis is needed to expand on the limited taxon sampling of prior morphological studies.

Given the limited taxon sampling of past comparative studies and the uncertainty of Štys' [44] taxon sampling, we investigated the presence of stridulatory mechanisms across a broad sample of Coreoidea. Our taxon sampling strategy aimed to sample species in all five families, as well as across many subfamilies, tribes and genera. We first conducted a morphological survey of stridulatory mechanisms known to occur in some species of Coreoidea, while also surveying for others not reported in coreoids but otherwise known to occur in other species of Heteroptera. Based on the results of our comparative morphological study, we then inferred a phylogeny of the superfamily (excluding the Stenocephalidae [one genus, 30 species] and Hyocephalidae [two genera, three species] due to lack of suitable tissues) using a large phylogenomic dataset. Our resulting phylogenetic hypotheses were used to study the evolution of stridulatory mechanisms using ancestral state estimation (ASE).

## 2. Material and methods

### 2.1. Comparative morphology

We examined 139 species for the presence of stridulatory mechanisms across all five families of the Coreoidea, as well as a species of the outgroup families Largidae and Pyrrhocoridae. We primarily surveyed for the presence of the three stridulatory mechanisms listed in table 1, when suitable material was available, and we also conducted a cursory survey for other stridulatory mechanisms known to occur in other families of Heteroptera (see [22]). When possible, males and females of each species were examined to evaluate if there is sexual dimorphism with respect to the presence of stridulatory mechanisms.

For all species, external morphology was examined using a Nikon SMZ1500 stereoscope. To view the posterolateral edge of the prothoracic foramen, we detached the head and pronotum from the remainder of the body. Similarly, we spread or detached the hemelytra and metathoracic wings from the body for examination. Prior to scanning electron microscopy (SEM), specimens of 29 species were coated with gold/palladium with a Cressington 108 Auto sputter coater and a Cressington MTM 20 Thickness controller. Structures were then imaged with a Tescan Vega 3xm SEM under high vacuum.

### 2.2. Molecular methods and sequence alignment

We retrieved published UCE sequence capture data for 205 taxa [50–53,55] (electronic supplementary material, table S1). We generated new sequence data for an additional 16 taxa (electronic supplementary material, table S1), which—compared to prior phylogenomic studies—included an expanded sampling of species within the tribes Coreini, Gonocerini and Phyllomorphini. We also included the monotypic Prionotylini, which is the first time this tribe has been included in a phylogenetic analysis. Genome sequences of *Halyomorpha halys* (Stål, 1855) and *Oncopeltus fasciatus* (Dallas, 1852) were also downloaded from NCBI to extract UCE sequences from scaffolds. Of the 221 taxa sampled for UCE data, 114 were also included in the above comparative morphological survey.

For our 16 newly sampled taxa, we used freshly preserved samples in ethanol (EtOH), except for one specimen pinned dried (electronic supplementary material, table S2). Genomic DNA was extracted either from the whole body or any part of the body (e.g. thorax, abdomen and/or legs) to sample similar amounts of tissue across samples. For the 15 samples preserved in EtOH, DNA was extracted using a Qiagen DNeasy Blood and Tissue kit (DNeasy) with the following modifications to the manufacturer's protocol: tissue was incubated for 24 h in a solution of 180 µl Buffer ATL and 20 µl proteinase K, and DNA was eluted twice with 50 µl Buffer AE. For the pinned sample, DNA was extracted with a DNeasy kit coupled with a Qiagen QIAquick PCR purification kit (‘DNQIA’; [51,52,56]). The DNQIA protocol initially followed the DNeasy protocol described above, but a QIAquick spin column was used, the AW washes were replaced with Buffer PE, and the DNA was eluted twice with 50 µl Buffer EB.

We assessed DNA quality and quantity using 1% agarose gel electrophoresis and a Qubit 2.0 fluorometer, respectively. Where possible, samples were subsequently normalized (10–20 ng/µl), and high molecular weight samples were fragmented into 200–1000 bp using a Covaris M220 Focused-ultrasonicator (20–60 s). For DNA extracted from the pinned specimen, DNA was repaired with a PreCR Repair Mix kit, followed by a 3X SPRI clean-up.

Libraries were made using a modified KAPA Hyper Prep Kit protocol, with all steps using half volume reactions. DNA samples were ligated to iTru universal adapter stubs and 8 bp dual indexes [57]. Library amplification was done via initial denaturation at 98°C for 3 min; followed by 14–16 cycles of 98°C for 30 s, 60°C for 30 s and 72°C for 30 s; and a final extension at 72°C for 5 min. The quality and quantity of amplified libraries were then inspected by gel electrophoresis and Qubit, respectively. Libraries were then combined into 1000 ng pools in equimolar amounts, dried at 60°C and resuspended in 14 µl IDTE.

Target enrichment was done using baits designed from two pentatomomorphan taxa [50,58] and a hybridization mixture with 1/2 volume of baits for samples derived from fresh material and 1/4 volume of baits for samples derived from pinned material. Baits were hybridized with libraries at 65°C for 12 h followed by 62°C for 12 h and then 60°C for 12 h, following Forthman *et al*.'s [52] touchdown capture protocol. Dynabeads M-280 Streptavidin beads were bound to bait-target hybrids, washed four times at 60°C and resuspended in 30 µl IDTE. The post-capture PCR amplification mix included 2.5 µl each of 5 µM iTru P5/P7 primers [57], and 14–18 cycles of post-capture amplification were performed following manufacturer's protocol, except an annealing temperature of 60°C and an extension period of 45 s were used. Post-amplification clean-up used Hydrophobic Sera-Mag SpeedBeads Carboxyl Magnetic Beads, followed by two washes in 70% EtOH. Enriched library pools were resuspended in 22 µl IDTE, quantified with Qubit and pooled into a single pool in equimolar amounts for sequencing on a single Illumina HiSeq3000 lane (2 × 100) at the University of Florida's Interdisciplinary Center for Biotechnology Research.

Unless otherwise stated, default settings were used for all data processing steps described below. Adapters were trimmed from demultiplexed, raw sequence reads with illumiprocessor v. 2.0 [59,60]. Duplicate reads were excluded using PRINSEQ-lite v. 0.20.4 [61], and the remaining reads were error-corrected with QuorUM v. 1.1.0 [62]. Reads were then *de novo* assembled with SPAdes v. 3.13.0 using the single-cell and auto coverage cut-off options [63]. PHYLUCE v. 1.7.0 [64] was used to identify and extract UCE loci from assembled contigs following Forthman *et al*. [50–52]. We also used PHYLUCE to align UCE baits to the *H. halys* and *O. fasciatus* genome sequences and to extract UCE loci with 500 bp of flanking nucleotides. Loci were aligned individually using PHYLUCE's implementation of MAFFT [65,66] and the following settings: generate incomplete matrices (--incomplete-matrix), no alignment trimming (--no-trim) and allow nucleotide uncertainty (--ambiguous). Locus alignments were then trimmed using trimAl v. 1.2 [67] and the heuristic automated1 method. We selected locus alignments with at least 50%, 65% and 80% of the total taxa (referred to as ‘50p’, ‘65p’ and ‘80p’ datasets, respectively). We also subsampled each dataset for the 25% most parsimony-informative (25mi) loci. As a result of our data filtering strategies, we generated six datasets for phylogenetic analyses. A summary of newly generated read, contig and UCE data are given in electronic supplementary material, table S2, and a summary of our alignments are presented in electronic supplementary material, table S3.

### 2.3. Phylogenetic inference

For each dataset, we concatenated locus alignments with PHYLUCE. Loci were initially treated as individual partitions, and the best model of sequence evolution and partitioning scheme were determined using IQ-Tree v. 2.1.2 [68] with the following settings: -m MF + MERGE [69], -rcluster 10 and -mrate E,I,G,R (i.e. I + G excluded from model selection since these parameters are not independent of each other [70,71]). Five partitioned maximum-likelihood (ML) analyses [72] were then performed in IQ-Tree for each dataset with the following settings: keep identical sequences (--keep-identical), remove partitions violating stationarity and homogeneity assumptions (--symtest-remove-bad; [73]), 1000 ultrafast bootstrap replicates further optimized by nearest neighbour interchange based on bootstrap alignments (-B 1000 -bnni; [74]) and 1000 Shimodaira-Hasegawa-like approximate likelihood ratio test replicates (-alrt 1000; [75]). The phylogeny with the best log-likelihood was then selected for each dataset.

Because gene tree discordance can result in concatenation methods supporting an incorrect species tree under high levels of incomplete lineage sorting, we also inferred species trees under the multispecies coalescent (MSC) model [76–79]. For each locus alignment, IQ-Tree was used to select the best-fit model of sequence evolution (-mrate E,I,G,R) and subsequently infer the gene tree (-m MFP) with near-zero length branches collapsed (-czb). We then excluded gene trees whose loci violated stationarity and homogeneity assumptions. The remaining optimal gene trees were then used to infer the species tree using ASTRAL-III v5.7.7 [80–82]. Clade support was assessed using local posterior probabilities [81].

We computed a 50% majority rule consensus tree based on all our ML and MSC estimated phylogenies in PAUP* v4.0a169 [83] to quickly assess overall congruence among analyses.

### 2.4. Ancestral state estimation

Due to the limitations of our phylogenetic taxon sampling (i.e. few representatives of Rhopalidae), we restricted ASE of stridulatory mechanisms to the Coreidae + Alydidae clade. Because only two stridulatory mechanisms were observed in this clade, we coded a single, multistate character with the following states: (0) mechanisms absent, (1) prothorax–hemelytron mechanism and (2) hemelytron–metafemur mechanism. Prior to ASE, we first used IQ-Tree to estimate branch lengths in units of substitutions on the MSC trees. We then pruned outgroup and rhopalid taxa from all ML and MSC phylograms. We also pruned alydid and coreid taxa with missing stridulatory mechanism data with two exceptions: 1) when one sex was observed to have a stridulatory mechanism but the other sex was not available for observation, we retained the taxon and coded it as having that mechanism; and 2) when taxa sampled in the comparative study were not included in the phylogeny but had a congener with data or with partial data, we retained the congener and coded it as having the same state as the corresponding taxon from the comparative study. We generated ultrametric trees using the *chronos* function in the *ape* v. 5.6.1 package [84] with R v. 4.1.2 [85]. We calibrated the root node to a relative age of 1. Four models (correlated, discrete, relaxed, clock) with three different lambda values (0.1, 1, 10) were tested. We selected the ultrametric tree with the highest penalized log-likelihood value for ASE.

We performed ASE using marginal reconstruction with the *rayDISC* function in the R package *corHMM* v. 2.7 [86]. We tested three models (equal rates, symmetric rates and all rates different) and compared the Akaike Information Criterion corrected for small sample size (AICc) [87]. If AICc values between models differed by greater than 2, we selected the model with the lowest AICc, which corresponds to a better fitting model. When AICc values differed by ≤2, we chose the simplest model.

## 3. Results

### 3.1. Occurrence of stridulatory mechanisms across Coreoidea

**Figure 2.**
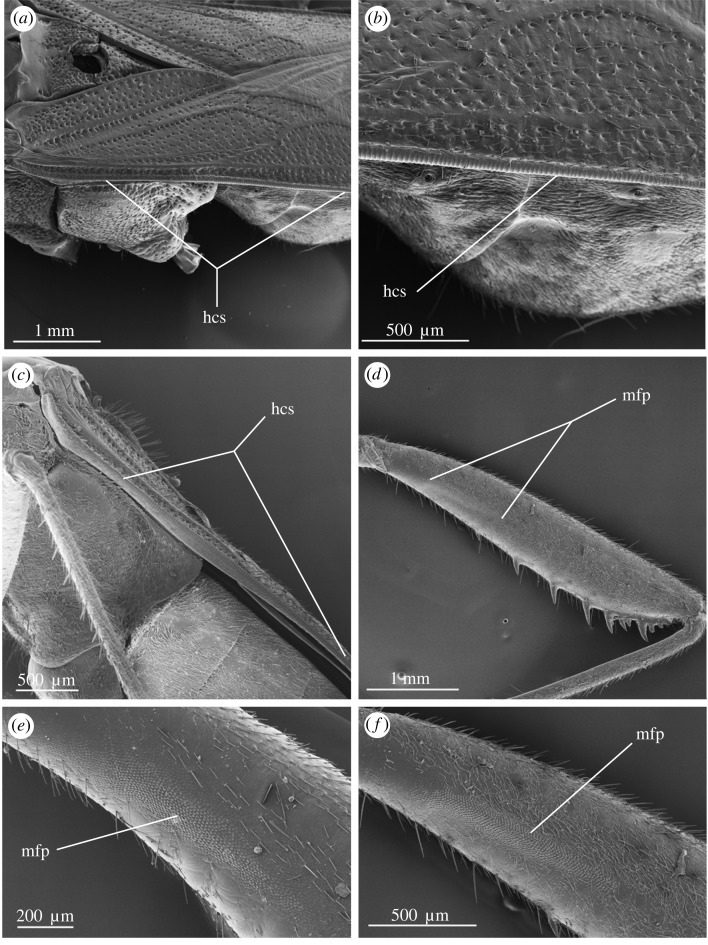
Scanning electron microscopy (SEM) image of the hemelytron–metafemur stridulatory mechanism in the Alydidae. (*a*) *Alydus calcaratus*, stridulitrum on hemelytron, male, dorsolateral view. (*b*) Close-up view of stridulitrum on hemelytron of *Alydus calcaratus*, male, dorsolateral view. (*c*) *Tollius curtulus*, stridulitrum on hemelytron, female, lateral view. (*d*) *Tollius curtulus*, plectrum on metafemur, male, posterior view. (*e*) *Alydus calcaratus*, plectrum on metafemur, male, posterior view. (*f*) Close-up view of plectrum on metafemur of *Tollius curtulus*, male, posterior view. hcs, hemelytral costal stridulitrum; mfp, metafemoral plectrum.

Of the 139 species examined in our morphological survey across all five families of Coreoidea, 31 species (22 genera) in three families (six tribes and four subfamilies) had putative stridulatory mechanisms. We confirmed the presence of three stridulatory mechanisms previously reported in some species of the Coreoidea (table 3), and we did not find any evidence of sexual dimorphism in the presence of these mechanisms. The hemelytron–metafemur mechanism was restricted to five species in four examined genera of the subfamily Alydinae (Alydidae) (out of 16 species in 12 genera) (table 3). As previously described, the costal margin of the hemelytron is modified into a stridulitrum (figure 2*a–c*), with the basal region of the posterior metafemoral surface possessing a fine patch of tubercles that forms the plectrum (figure 2*d–f*).

**Table 3.** Taxonomic distribution of four stridulatory mechanisms in the Coreoidea and some outgroup taxa. Cells with + symbol indicate presence of mechanism, cells with — symbol indicate absence of mechanism, and cells with NE or NA indicate a mechanism could not be examined or is not applicable, respectively. Cu, cubitus vein; F, female; M, male; Sc+R, subcosta + radius vein.

taxon	prothorax−hemelytron		hemelytron−metafemur		metawing Sc+R−abdomen		metawing Cu−thorax/abdomen	
	M	F	M	F	M	F	M	F
**ALYDIDAE Amyot & Serville, 1843**								
ALYDINAE Amyot & Serville, 1843								
*Alydus calcaratus* (Linnaeus, 1758)	−	−	**+**	**+**	−	−	−	−
*Alydus pilosulus* Herrich-Schäffer, 1847	−	−	**+**	**+**	−	−	−	−
*Apidaurus conspersus* Stål, 1870	−	NE	−	NE	−	NE	−	NE
*Burtinus notatipennis* (Stål, 1860)	−	−	**+**	**+**	−	−	−	−
*Camptopus lateralis* (Germar, 1817)	−	−	−	−	−	NE	−	NE
*Hamedius incarnatus* (Erichson, 1842)	−	NE	−	−	−	NE	−	NE
*Hyalymenus longispinus* Stål, 1870	−	NE	−	NE	NE	NE	NE	NE
*Hyalymenus subinermis* Van Duzee, 1923	−	−	−	−	−	−	−	−
*Hyalymenus* sp.	−	NE	−	NE	−	NE	−	NE
*Megalotomus quinquespinosus* (Say, 1825)	−	−	**+**	**+**	−	−	−	−
*Melanacanthus scutellaris* (Dallas, 1852)	−	NE	−	NE	−	NE	−	NE
*Neomegalotomus rufipes* (Westwood, 1842)	NE	−	NE	−	NE	−	NE	−
*Neomegalotomus parvus* (Westwood, 1842)	−	−	−	−	−	−	−	−
*Riptortus pedestris* (Fabricius, 1775)	NE	−	−	−	NE	−	NE	−
*Stachyocnemus apicalis* (Dallas, 1852)	−	−	−	−	−	−	−	−
*Tollius curtulus* (Stål, 1859)	−	NE	**+**	NE	−	NE	−	NE
MICRELYTRINAE Stål, 1868								
**Leptocorisini Stål, 1872**								
*Leptocorisa acuta* (Thunberg, 1783)	−	−	−	−	−	−	−	−
*Stenocoris tipuloides* (De Geer, 1773)	−	NE	−	−	−	−	−	−
**Micrelytrini Stål, 1868**								
*Dulichius trispinosus* Stål, 1866	−	−	−	−	−	−	−	−
*Micrelytra fossularum* (Rossi, 1790)	−	−	−	−	NA	NA	NA	NA
**COREIDAE Leach, 1815**								
COREINAE Leach, 1815								
**Acanthocephalini Stål, 1870**								
*Acanthocephala declivis* (Say, 1832)	−	−	−	−	−	−	−	−
*Acanthocephala femorata* (Fabricius, 1775)	−	−	−	−	−	−	−	−
*Meluchopetalops banausus* Breddin, 1903	−	−	−	−	−	−	−	−
**Acanthocerini Bergroth, 1913**								
*Acanthocerus crucifer* Palisot de Beauvois, 1818	−	−	−	−	−	−	−	−
*Athaumastus haematicus* (Stål, 1860)	−	−	−	−	−	−	−	−
*Crinocerus sanctus* (Fabricius, 1775)	−	−	−	−	−	−	−	−
*Euthochtha galeator* (Fabricius, 1803)	−	−	−	−	−	−	−	−
*Zoreva lobulata* Stål, 1870	−	−	−	−	−	−	−	−
**Acanthocorini Amyot & Serville, 1843**								
*Acanthocoris sordidus* (Thunberg, 1783)	−	−	−	−	−	−	−	−
*Physomerus grossipes* (Fabricius, 1794)	−	−	−	−	−	−	−	−
*Rhyticoris terminalis* (Burmeister, 1835)	−	−	−	−	NE	−	NE	−
**Anisoscelini Laporte, 1832**								
*Chondrocera laticornis* Laporte, 1832	−	−	−	−	−	−	−	−
*Dalmatomammurius vandoesburgi* (Brailovsky, 1982)	−	−	−	−	−	−	−	−
*Diactor bilineatus* (Fabricius, 1803)	−	NE	−	−	−	NE	−	NE
*Leptoglossus occidentalis* Heidemann, 1910	−	−	−	−	−	−	−	−
*Leptoglossus phyllopus* (Linnaeus, 1767)	−	−	−	−	−	−	−	−
*Leptoglossus zonatus* (Dallas, 1852)	−	−	−	−	−	−	−	−
*Leptoscelis quadrisignatus* (Distant, 1881)	−	−	−	−	−	−	−	−
*Narnia femorata* Stål, 1862	−	−	−	−	−	−	−	−
*Narnia snowi* Van Duzee, 1906	−	−	−	−	−	−	−	−
*Phthiacnemia picta* (Drury, 1773)	−	−	−	−	−	NE	−	NE
**Chariesterini Stål, 1868**								
*Chariesterus antennator* (Fabricius, 1803)	−	−	−	−	NE	NE	NE	NE
*Chariesterus armatus* (Thunberg, 1825)	−	−	−	−	−	−	−	−
*Plapigus circumcinctus* Stål, 1860	−	−	−	−	NE	−	NE	**−**
**Chelinideini Blatchley, 1926**								
*Chelinidea tabulata* (Brumeister, 1835)	−	−	−	−	−	−	−	−
*Chelinidea vittiger* Uhler, 1863	−	−	−	−	−	−	−	−
**Cloresmini Stål, 1873**								
*Cloresmus* sp.	−	−	−	−	−	−	−	−
**Colpurini Breddin, 1900**								
*Hygia* sp.	−	NE	−	NE	−	NE	−	NE
*Sciophyroides sulcicrus* (Breddin, 1900)	−	−	−	−	NE	NE	NE	NE
**Coreini Leach, 1815**								
*Centrocoris spiniger* (Fabricius, 1781)	**+**	**+**	**−**	**−**	NE	**−**	NE	**−**
*Centrocoris variegatus* Kolenati, 1845	**+**	**+**	−	−	−	−	−	−
*Coreus marginatus* (Linnaeus, 1758)	**+**	**+**	−	−	−	−	−	−
*Enoplops disciger* (Kolenati, 1845)	**+**	NE	−	−	−	NE	−	NE
*Enoplops scapha* (Fabricius, 1794)	**+**	**+**	−	−	NE	−	NE	−
*Haploprocta sulcicornis* (Fabricius, 1794)	NE	**+**	−	−	NE	−	NE	−
*Spathocera dalmanii* (Schilling, 1829)	NE	**+**	−	−	NE	−	NE	−
*Syromastus rhombeus* (Linnaeus, 1767)	**+**	**+**	−	−	−	−	−	−
**Daladerini Stål, 1873**								
*Dalader planiventris* (Westwood, 1842)	−	−	−	−	−	−	−	−
*Odontocurtus consociatus* Brailovsky, 2011	−	−	−	−	−	−	−	−
**Dasynini Bergroth, 1913**								
*Aulacosternum nigrorubrum* Dallas, 1852	−	−	−	−	−	NE	−	NE
Dasynini sp.	NE	−	NE	−	NE	NE	NE	NE
**Discogastrini Stål, 1868**								
*Discogaster dentipes* (Stål, 1868)	−	−	−	−	−	−	−	−
*Savius diagonalis* Berg, 1892	−	−	−	−	−	−	−	−
**Gonocerini Mulsant & Rey, 1870**								
*Cletoliturus lituripennis* (Stål, 1855)	**+**	**+**	−	−	−	−	−	−
*Cletomorpha benita* Kirby, 1891	**+**	NE	−	NE	−	NE	−	NE
*Cletomorpha nyasana* Bergroth, 1914	**+**	**+**	−	−	−	−	−	−
*Cletoscellus spinijugis* (Bergroth, 1905)	**+**	NE	−	NE	NE	NE	NE	NE
*Cletus binotulatus* (Stål, 1858)	NE	**+**	−	−	−	−	−	−
*Cletus ochraceus* (Herrich-Schäffer, 1840)	**+**	NE	−	NE	−	NE	−	NE
*Cletus poikilus* Brailovsky, 2011	**+**	NE	−	NE	−	NE	−	NE
*Cletus* sp.	**+**	**+**	−	−	−	−	−	−
*Gonocerus acuteangulatus* (Goeze, 1778)	−	−	−	−	NE	−	NE	−
*Gonocerus insidiator* (Fabricius, 1787)	−	−	−	−	−	−	−	−
*Plinachtus bicoloripes* Scott, 1847	−	NE	−	NE	−	NE	−	NE
**Homoeocerini Amyot & Serville, 1843**								
*Fracastorius cornutus* Distant, 1902	−	NE	−	NE	NE	NE	NE	NE
*Homoeocerus angulatus* Westwood, 1842	NE	−	NE	−	NE	NE	NE	NE
*Homoeocerus bipustulatus* Stål, 1871	NE	−	NE	−	NE	−	NE	−
*Homoeocerus marginellus* (Herrich-Schäffer, 1840)	−	−	−	−	−	−	−	−
*Prismatocerus auriculatus* Stål, 1866	−	−	−	−	−	−	−	−
**Hypselonotini Bergroth, 1913**								
*Acidomeria sordida* (Berg, 1879)	−	NE	−	NE	−	NE	−	NE
*Althos obscurator* (Fabricius, 1803)	−	−	−	−	−	−	−	−
*Anasa andresii* (Guérin-Méneville, 1857)	−	**−**	**−**	**−**	**−**	NE	**−**	NE
*Anasa tristis* (De Geer, 1773)	−	−	−	−	−	−	−	−
*Catorhintha guttula* (Fabricius, 1794)	−	−	−	−	−	−	−	−
*Catorhintha texana* Stål, 1870	−	−	−	−	−	NE	−	NE
*Cebrenis cauta* Brailovsky, 1995	−	−	−	−	−	−	−	−
*Cebrenis danieli* Brailovsky, 1995	−	−	−	−	−	NE	−	NE
*Cebrenistella robusta* (Stål, 1870)	−	−	−	−	−	−	−	−
*Hypselonotus fulvus* (De Geer, 1773)	−	−	−	−	−	−	−	−
*Hypselonotus punctiventris* Stål, 1862	−	−	−	−	−	−	−	−
*Scolopocerus secundarius* Uhler, 1875	NE	**+**	NE	−	NE	NE	NE	NE
*Scolopocerus uhleri* Distant, 1881	**+**	**+**	−	−	−	−	−	−
*Sethenira testacea* Spinola, 1837	−	−	−	−	−	−	−	−
*Villasitocoris inconspicuus* (Herrich-Schäffer, 1842)	−	−	−	−	−	−	−	−
*Zicca taeniola* (Dallas, 1852)	−	−	−	−	−	−	−	−
**Latimbini Stål, 1873**								
*Latimbus concolor* (Germar, 1838)	−	NE	−	−	NE	NE	NE	NE
**Mictini Amyot & Serville, 1843**								
*Anoplocnemis curvipes* (Fabricius, 1781)	−	−	−	−	−	−	−	−
*Elasmopoda alata* (Westwood, 1842)	−	−	−	−	−	−	−	−
*Mictis longicornis* Westwood, 1842	−	−	−	−	−	NE	−	NE
*Mygdonia tuberculosa* (Signoret, 1851)	−	−	−	−	−	−	−	−
**Nematopodini Amyot & Serville, 1843**								
*Mozena arizonensis* Ruckes, 1955	−	−	−	−	−	−	−	−
*Thasus neocalifornicus* Brailovsky & Barrera, 1994	−	−	−	−	−	−	−	−
**Phyllomorphini Mulsant & Rey, 1870**								
*Pephricus paradoxus* (Sparrman, 1777)	**+**	**+**	−	−	−	−	−	−
*Phyllomorpha laciniata* (Villers, 1789)	**+**	**+**	−	−	NE	NE	NE	NE
*Tongorma latreillii* (Guérin-Méneville, 1839)	**+**	**+**	−	−	NE	NE	NE	NE
**Placoscelini Stål, 1868**								
*Plaxiscelis pagana* (Burmeister, 1835)	−	−	−	−	NE	−	NE	−
*Stenoeurilla mesoamericana* Brailovsky & Barrera, 2019	−	NE	−	NE	−	NE	−	NE
**Prionotylini Puton, 1872**								
*Prionotylus brevicornis* (Mulsant & Rey, 1852)	**+**	**+**	−	−	NA	NA	NA	NA
**Spartocerini Amyot & Serville, 1843**								
*Sephina pustulata* (Fabricius, 1803)	−	−	−	−	−	−	−	−
*Spartocera batatas* (Fabricius, 1798)	−	−	−	−	−	−	−	−
HYDARINAE Stål, 1873								
*Hydara tenuicornis* (Westwood, 1842)	−	−	−	−	−	−	−	−
*Hydarella chiangdaoensis* Brailovsky, 1994	−	−	−	−	−	−	−	−
*Hydaropsis longirostris* (Hsiao, 1963)	−	NE	−	−	−	−	−	−
*Madura fuscoclavata* Stål, 1860	−	−	−	−	−	−	−	−
*Maduranoides chemsaki* Brailovsky, 1988	−	NE	−	−	NE	NE	NE	NE
MEROPACHYINAE Stål, 1868								
**Merocorini Stål, 1870**								
*Merocoris curtatus* McAtee, 1919	−	−	−	−	−	−	−	−
**Spathophorini Kormilev, 1954**								
*Paralycambes pronotalis* Brailovsky, 1998	NE	−	NE	−	NE	**−**	NE	**−**
PSEUDOPHLOEINAE Stål, 1868								
**Clavigrallini Stål, 1873**								
*Gralliclava horrens* (Dohrn, 1860)	**−**	**−**	**−**	**−**	**−**	**−**	**−**	**−**
**Pseudophloeini Stål, 1868**								
*Ceraleptus gracilicornis* (Herrich-Schäffer, 1835)	NE	**−**	**−**	**−**	NE	**−**	NE	**−**
*Ceraleptus obtusus* (Brullé, 1839)	**−**	NE	**−**	NE	NE	NE	NE	NE
*Coriomeris affinis* (Herrich-Schäffer, 1839)	**−**	NE	**−**	NE	**−**	NE	**−**	NE
*Coriomeris occidentalis* Dolling & Yonke, 1976	**−**	**−**	**−**	**−**	**−**	**−**	**−**	**−**
*Hoplolomia scabricula* Stål, 1873	**−**	NE	**−**	**−**	NE	NE	NE	NE
*Mevanidea spiniceps* (Signoret, 1861)	**−**	**−**	**−**	**−**	**−**	**−**	**−**	**−**
*Vilga sanctipauli* Dolling, 1977	**−**	**−**	**−**	**−**	NE	**−**	NE	**−**
**HYOCEPHALIDAE Bergroth, 1906**								
*Hyocephalus aprugnus* Bergroth, 1906	NE	**−**	NE	**−**	NE	NA	NE	NA
*Maevius indecorus* Stål, 1874	**−**	**−**	**−**	**−**	NA	NA	NA	NA
**RHOPALIDAE Amyot & Serville, 1843**								
RHOPALINAE Amyot & Serville, 1843								
**Chorosomatini Fieber, 1860**								
*Chorosoma schillingii* (Schilling, 1829)	**−**	**−**	**−**	**−**	**−**	**−**	**−**	**−**
**Harmostini Stål, 1873**								
*Aufeius impressicollis* Stål, 1870	**−**	**−**	**−**	**−**	**−**	**−**	**+**	**−**
*Harmostes reflexulus* (Say, 1832)	**−**	**−**	**−**	**−**	**−**	**−**	**+**	**−**
*Harmostes serratus* Fabricius, 1775	**−**	**−**	**−**	**−**	**−**	**−**	**+**	**−**
**Niesthreini Chopra, 1967**								
*Arhyssus barberi* Harris, 1942	**−**	**−**	**−**	**−**	**−**	**−**	**−**	**−**
**Rhopalini Amyot & Serville, 1843**								
*Corizus hyoscyami* (Linnaeus, 1758)	**−**	**−**	**−**	**−**	**−**	**−**	**−**	**−**
*Liorhyssus hyalinus* (Fabricius, 1794)	**−**	**−**	**−**	**−**	**−**	**−**	**−**	**−**
*Maccevethus errans* (Fabricius, 1794)	**−**	NE	**−**	**−**	**−**	**−**	**−**	**−**
SERINETHINAE Stål, 1873								
*Boisea rubrolineata* (Barber, 1926)	**−**	**−**	**−**	**−**	**−**	**−**	**−**	**−**
*Jadera haematoloma* (Herrich-Schäffer, 1847)	**−**	**−**	**−**	**−**	**+**	**+**	**−**	**−**
*Leptocoris* sp.	**−**	**−**	**−**	**−**	**−**	**−**	**−**	**−**
**STENOCEPHALIDAE Dallas, 1852**								
*Dicranocephalus haoussa* (Villiers, 1950)	**−**	**−**	**−**	**−**	**−**	**−**	**−**	**−**
**LARGIDAE Amyot & Serville, 1843**								
LARGINAE Amyot & Serville, 1843								
**Largini Amyot & Serville, 1843**								
*Largus* sp.	**−**	**−**	**−**	**−**	**−**	**−**	**−**	**−**
**PYRRHOCORIDAE Dohrn, 1859**								
*Dysdercus suturellus* (Herrich-Schäffer, 1842)	**−**	**−**	**−**	**−**	**−**	**−**	**−**	**−**

**Figure 3.**
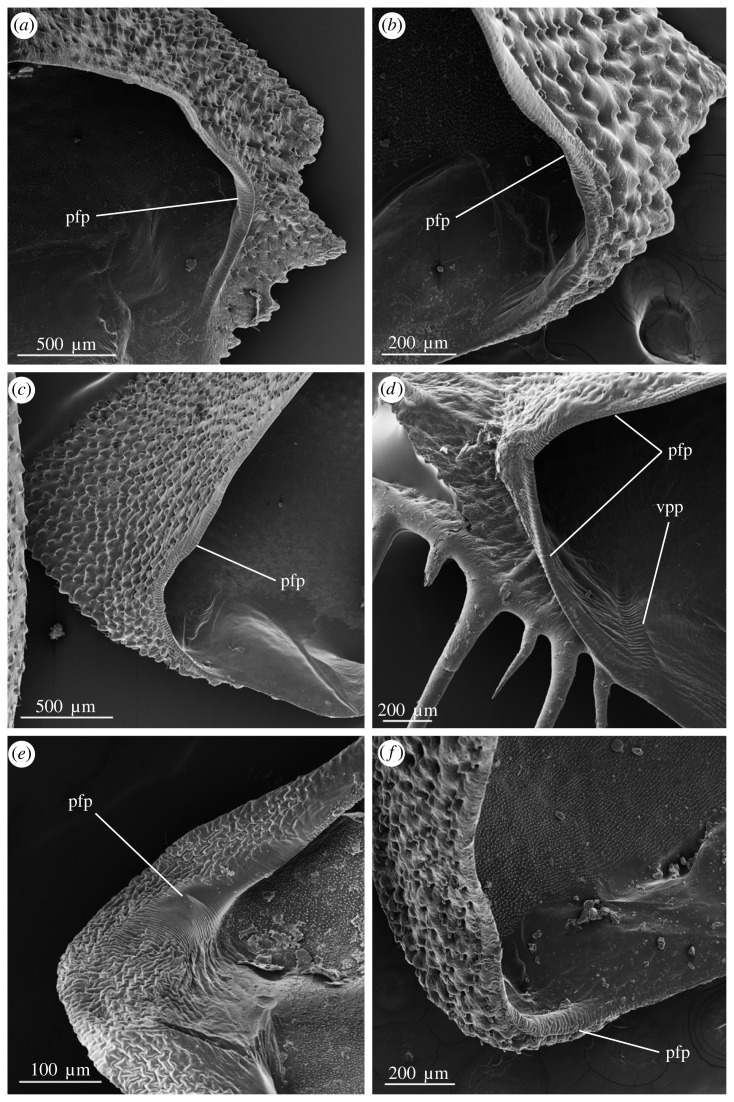
SEM image of the pronotal plectrum in the Coreidae, ventral view. (*a*) *Centrocoris spiniger*, male. (*b*) *Cletomorpha nyasana*, male. (*c*) *Coreus marginatus*, male. (*d*) *Pephricus paradoxus*, female. (*e*) *Prionotylus brevicornis*, female. (*f*) *Scolopocerus uhleri*, female. pfp, plectrum on ventrolateral foramen of prothorax; vpp, ventral prothoracic plectrum.

**Figure 4.**
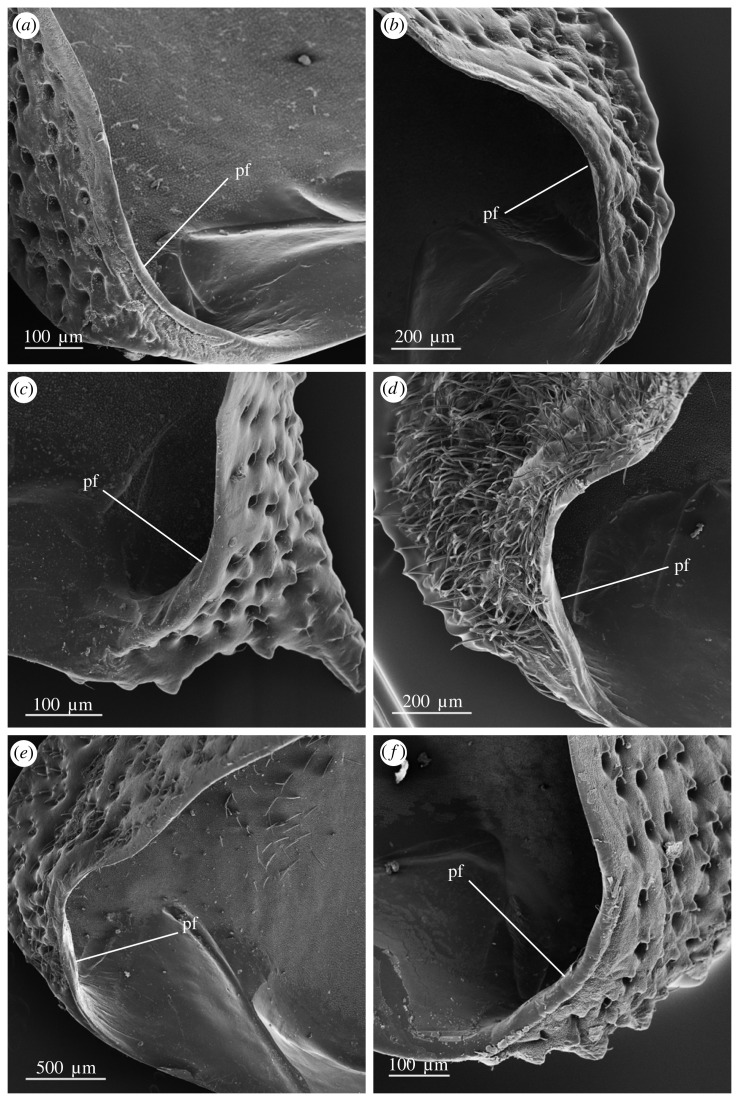
SEM image of the prothoracic ventrolateral foramen in the Coreidae, ventral view. (*a*) *Catorhintha guttula*, female. (*b*) *Euthochtha galeator*, male. (*c*) *Hydarella chiangdaoensis*, female. (*d*) *Leptoglossus phyllopus*, female. (*e*) *Rhyticoris terminalis*, female. (F) *Zicca taeniola*, female. pf, prothoracic foramen.

The prothorax–hemelytron stridulatory mechanism was only found within the Coreidae and was restricted to 22 species in 15 genera (five tribes) within the subfamily Coreinae (out of 90 species, 70 genera, 22 tribes) (table 3). For all these species, the plectrum was observed on the ventrolateral edge of the prothoracic foramen (figure 3). Species of the Phyllomorphini exhibited a far more extensive plectrum. In this tribe, the plectrum extended from near the prolegs to the paramedian part of the posterior prothoracic margin where it then extended inwards for about a quarter of the length of the pronotum (figure 3*d*). All other species of Coreidae examined lacked the prothoracic plectrum (figure 4).

**Figure 5.**
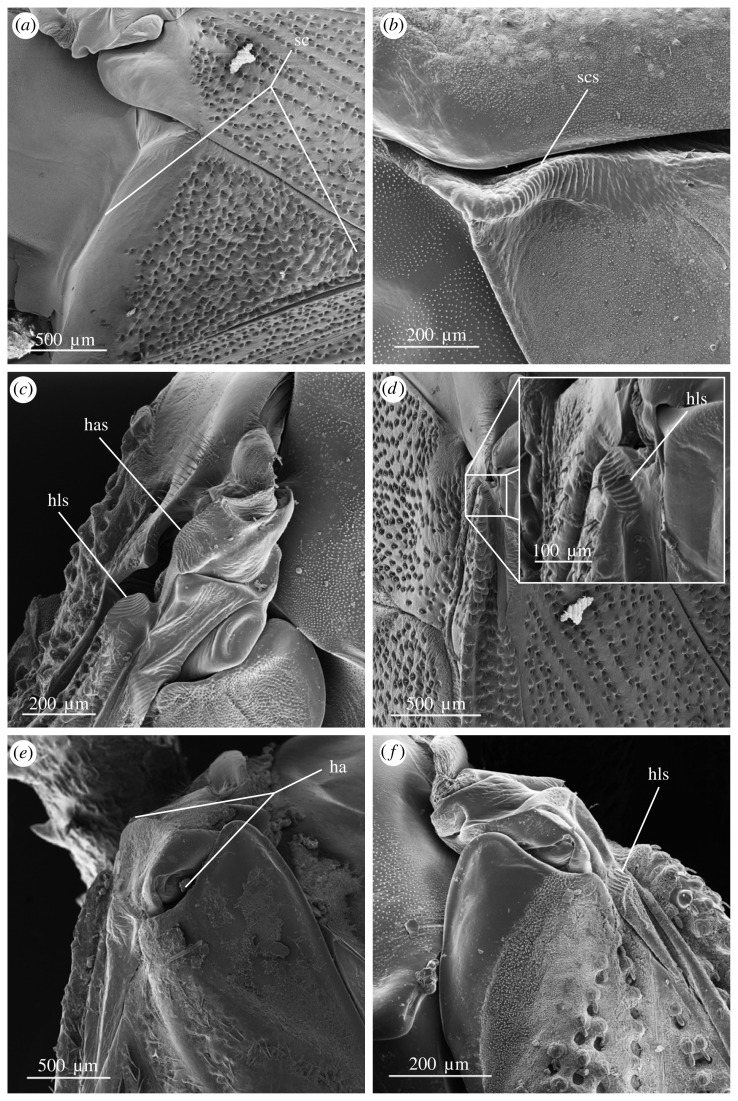
SEM image of the scutellum (*a,b*) and hemelytral axillary sclerites (*c–f*) in the Coreidae. (*a*) *Coreus marginatus*, scutellum, male, dorsal view. (*b*) *Pephricus paradoxus*, scutellum, female, dorsal view. (*c*) *Centrocoris spiniger*, axillary sclerites of hemelytron, male, dorsolateral view. (*d*) *Coreus marginatus*, axillary sclerites of hemelytron, male, dorsolateral view. (*e*) *Rhyticoris terminalis*, axillary sclerites of hemelytron, female, dorsolateral view. (*f*) *Scolopocerus uhleri*, axillary sclerites of hemelytron, male, dorsolateral view. ha, hemelytral axillary sclerites; has, stridulitrum of hemelytral axillary sclerites; hls, stridulitrum of hemelytral hypocostal lamina; sc, scutellum; scs, scutellar stridulitrum.

In species with the prothoracic plectrum, the stridulitrum was observed on one or more axillary sclerites of the hemelytron, often involving the hypocostal lamina (figure 5*c–f*). An additional stridulitrum was observed in the Phyllomorphini, which involved the lateral margins at the base of the scutellum (figure 5*b*) that was otherwise not present in all other taxa (figure 5*a*).

**Figure 6.**
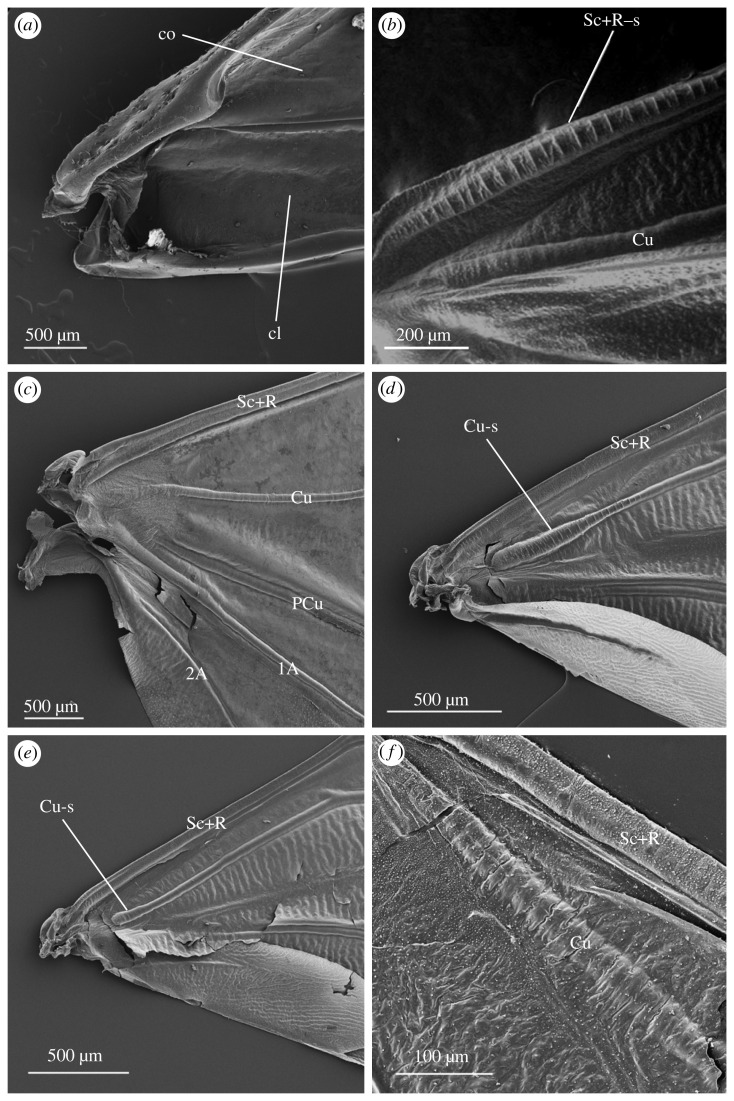
SEM image of the ventral surfaces of hemelytron (*a*) and metathoracic wings (*b–f*) in the Coreoidea. (*a*) *Rhyticoris terminalis*, hemelytron, female. (*b*) *Jadera haematoloma*, close-up of subcosta+radius and cubitus on metathoracic wing, male (reproduced and modified from Zych *et al*. [47] [their Fig. 4], Entomological Society of America). (*c*) *Euthochtha galeator*, proximal area of metathoracic wing, male. (*d*) *Harmostes serratus*, proximal area of metathoracic wing, male. (*e*) *Harmostes serratus*, proximal area of metathoracic wing, female. (*f*) *Dulichius trispinosus*, close-up of subcosta + radius and cubitus on metathoracic wing, male. cl, clavus; co, corium; Cu, cubitus; Cu-s, cubitus stridulitrum; PCu, postcubitus; Sc+R, subcosta + radius; Sc+R-s, subcosta + radius stridulitrum; 1A, first anal vein; 2A, second anal vein.

Our results did differ from published studies. We did not observe a prothorax–hemelytron stridulatory mechanism in *Euthochtha galeator* (Fabricius, 1803) (Acanthocerini) (figure 4*b*). We also did not confirm the ventral surface of the clavus and the metathoracic wing mechanism for *Rhyticoris* Costa, 1863 (figure 6*a*).

We confirmed the presence of the metathoracic wing-abdomen mechanism in *Jadera haematoloma* (figure 5*b*), and in another surveyed congener. In this genus, the stridulitrum is located on the Sc+R vein of the metathoracic wing. The plectrum in *Jadera* Stål, 1862 involves an anterior-posterior movement of the abdominal tergal plate (abdominal tergites I and II) (as experimentally shown by Zych *et al*. [47]). We did not observe this mechanism in any other serinethine genera (i.e. *Boisea* Kirkaldy, 1910 and *Leptocoris* Hahn, 1833) or in any species of Rhopalinae.

**Figure 7.**
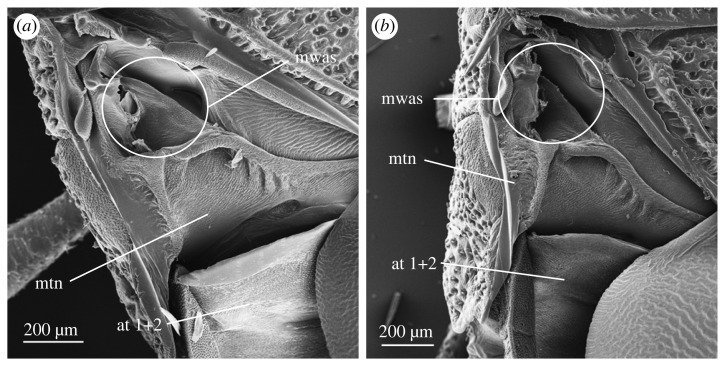
SEM image of the metathorax and abdomen in the Rhopalidae, dorsal view. (*a*) *Harmostes serratus*, male. (*b*) *Harmostes serratus*, female. at 1+2, abdominal tergites 1 and 2; mtn, metanotum; mwas, metathoracic wing attachment site.

We additionally found the presence of a fourth stridulatory mechanism not previously documented in the Coreoidea. Within the tribe Harmostini (Rhopalidae: Rhopalinae), a stridulitrum was observed on the cubitus (Cu) vein of the metathoracic wing in species of *Harmostes* Burmeister, 1835 and *Aufeius* Stål, 1870 (table 3; Figures 1, 6*d*, 6*e*). In all species examined of this tribe, we also observed sexual dimorphism in the presence of the stridulitrum; it was always present in males (figurer 6*d*) and absent or drastically reduced to a couple of barely elevated transverse ridges in females (table 3; figure 6*e*). While a distinct plectrum was not obvious on the metanotum and/or abdomen (figure 7), it is possible a similar mechanism reported by Zych *et al*. [47] for *J. haematoloma* occurs in these species.

No other stridulatory mechanisms were observed in our taxon sampling. Furthermore, we did not find any evidence of stridulatory mechanisms occurring in the coreoid families Hyocephalidae and Stenocephalidae (table 3).

### 3.2. Phylogeny of Coreoidea

**Figure 8.**
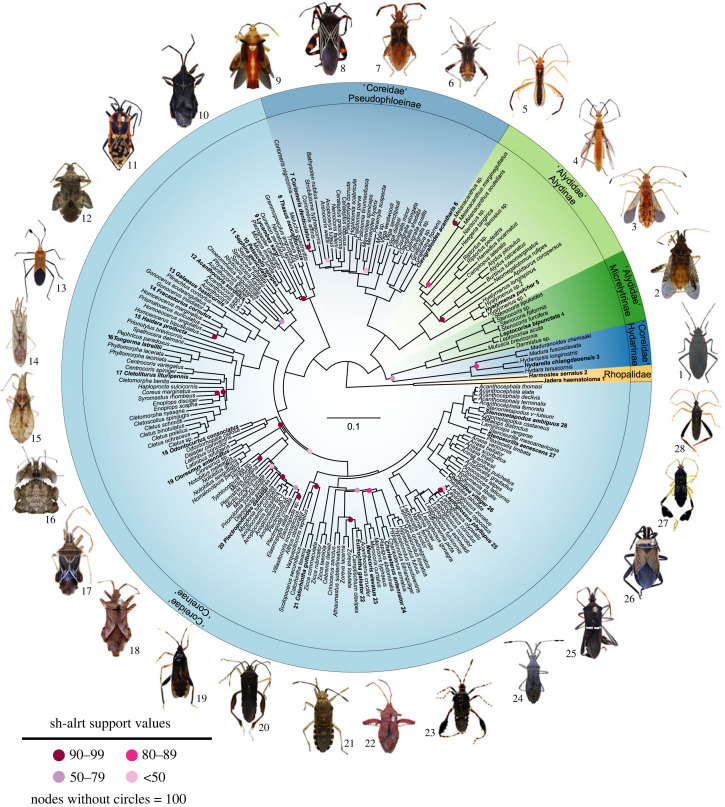
Maximum likelihood (ML) best tree of the Coreoidea based on the 50p concatenated ultraconserved element alignment. For visualization, outgroup taxa are pruned from the tree. Circles at nodes represent Shimodaira-Hasegawa-like approximate likelihood ratio test (sh-alrt) support; nodes without circles have 100% support (see Data Availability for tree with all terminals and sh-alrt and ultrafast bootstrap values visible). Dorsal habitus images of select representatives (not to scale) are given to show a range of diversity within the Coreoidea (terminal taxa with images bolded and have corresponding numbers associated with images).

Our analyses were congruent in many of the phylogenetic relationships they recovered (figure 8, electronic supplementary material, figure S1), with most nodes moderately to highly supported (figure 8; see tree files in Data Availability). However, the 50% majority rule consensus tree revealed much conflict regarding the positions of the Colpurini, Cloresmini, Daladerini + Latimbini clade, Mictini and Acanthocephalini + Acanthocerini + Anisoscelini + Chariesterini + Chelinideini + Hypselonotini + Merocorini + Placoscelini clade (electronic supplementary material, figure S1), which was largely caused by the dubious position of *Elasmopoda alata* (Westwood, 1842) across most analyses (see tree files in Data Availability); when looking across all phylogenetic results, we consistently recovered (Daladerini + Latimbini) as sister to Cloresmini + (Colpurini + Mictini [with or without *Elasmopoda alata*]). Overall, the higher-level relationships we recovered are congruent with previous phylogenomic results [50–54], including the paraphyletic natures of the Alydidae and Coreidae. However, our improved taxon sampling highlighted some new and notable relationships. First, we found the Prionotylini to be sister to a species of Coreini, within a large clade comprised of the Coreini, most Gonocerini and Phyllomorphini (figure 8, electronic supplementary material, figure S1). Secondly, while we supported a polyphyletic Gonocerini as in previous phylogenomic studies, we found a small clade including *Gonocerus* Berthold, 1827 and *Plinachtus* Stål, 1860 (genera not included in prior UCE studies) to form a sister group relationship with the Homoeocerini (figure 8, electronic supplementary material, figure S1).

### 3.3. The evolution of stridulatory mechanisms

**Figure 9.**
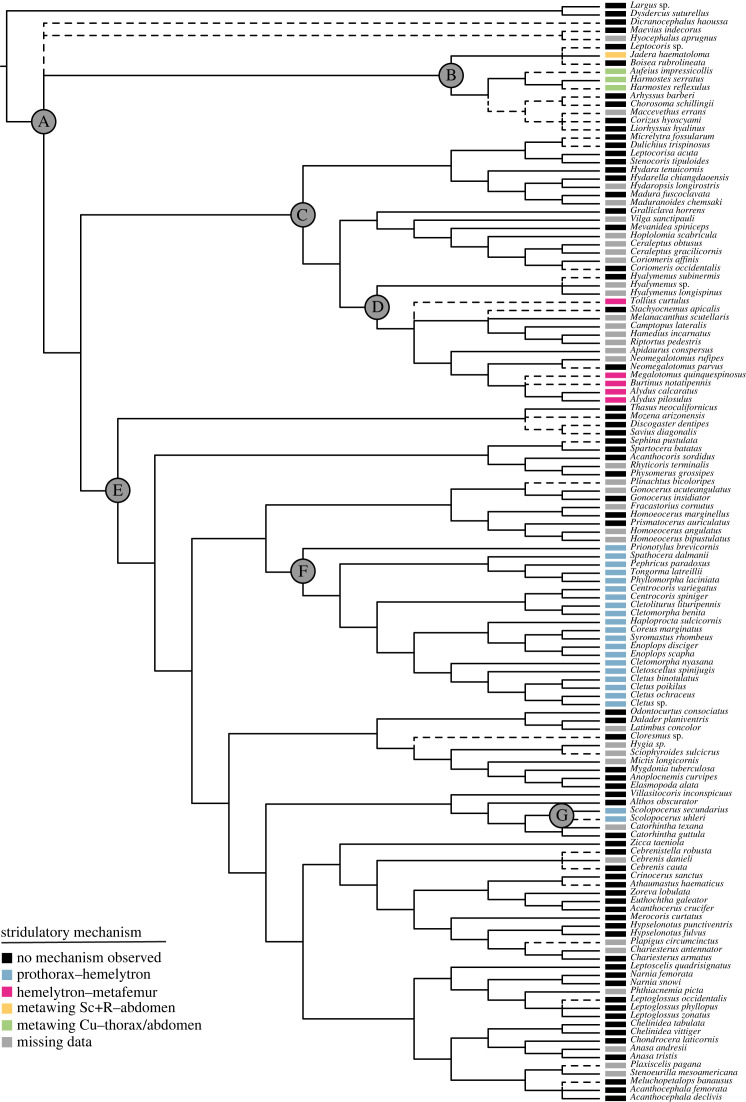
Character states of observed stridulatory mechanisms displayed for terminal taxa on the right. Cladogram is based on the 50p ML best tree (figure 8), but taxa with missing data are pruned. Dashed branches represent taxa not included in phylogenetic analyses but that are inserted into the tree based on current coreoid classification and recent phylogenomic studies to allow inclusion of additional taxa where presence or absence of stridulatory mechanisms are known. Labels at select nodes refer to nodes that are discussed in the text. Cu, cubitus; Sc+R, subcosta + radius.

Plotting the occurrence of stridulatory mechanisms within the Coreoidea on our phylogenetic hypothesis (figure 9, Node A)—after inserting taxa from the comparative study that were not included in phylogenetic analysis based on our current understanding of coreoid phylogeny and taxonomy—shows that two mechanisms were only found in the Rhopalidae (Node B), while the ‘Alydidae’ (Node C) and ‘Coreidae’ (Node E) each had their own distinctive mechanism. Within the Rhopalidae, we found the metawing Cu-thorax/abdomen mechanism to be a likely synapomorphy of the rhopalid tribe Harmostini. We also found the metawing Sc+R-abdomen mechanism to be autapomorphic for the rhopalid genus *Jadera*. The hemelytron–metafemur stridulatory mechanism within the Alydinae (Node D) is primarily found in a single clade of three genera; the mechanism was also observed in *Tollius* Stål, 1870, but the phylogenetic position of this genus remains uncertain, and thus, whether this mechanism is a potential non-homoplastic synapomorphy remains unknown. Within the ‘Coreidae’, the prothorax–hemelytron mechanism was observed in two clades; the majority of coreid species with this mechanism formed a large clade (Clade F), with only *Scolopocerus* Uhler, 1875 forming a separate clade (Clade G).

**Figure 10.**
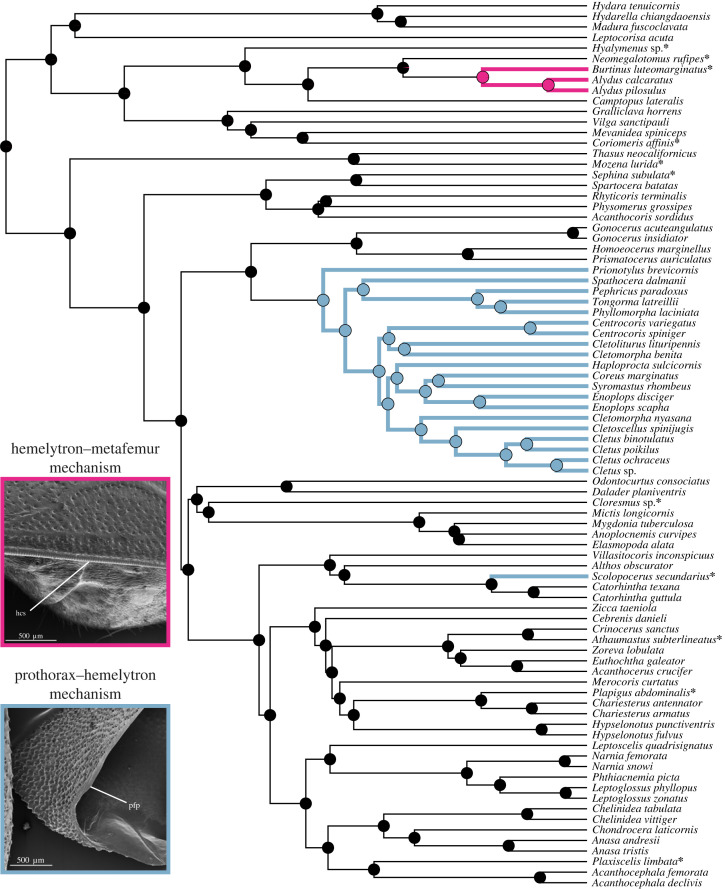
Ancestral state estimates (ASE) of two stridulatory mechanisms observed in the Alydidae and Coreoidea based on the 50p ML ultrametric tree. Taxa with missing data are pruned from the tree for analysis. Taxa not included in the phylogenetic study are also excluded. Terminal taxa with asterisks indicate character states were coded for these taxa based on a congener from the comparative study (see Methods for more details). Pie charts at nodes show the probability of stridulatory mechanism conditions, with branches similarly coloured to represent the most likely state. SEM image of the plectrum or stridulitrum associated with the stridulatory mechanisms included in the ASE analysis. hcs, hemelytral costal stridulitrum; pfp, plectrum on ventrolateral foramen of prothorax.

Despite some topological incongruences across our phylogenetic hypotheses, ASE analyses of stridulatory mechanisms within the Alydidae and Coreidae were congruent (figure 10, electronic supplementary material, figure S2–S12), based on the best fitting equal rates model. The common ancestors of the Coreidae + Alydidae, ‘Alydidae’ and ‘Coreidae’ did not possess the prothorax–hemelytron or the hemelytron–metafemur stridulatory mechanisms. The presence of stridulatory mechanisms, in general, was estimated to have evolved independently at least three times in the Alydidae + Coreidae clade, and, based on our results, these mechanisms have not been lost in descendant branches. The common ancestor of *Burtinus* Stål, 1860 and *Alydus* Fabricius, 1803 was estimated to possess the hemelytron–metafemur mechanism. Our results also supported the presence of the prothorax–hemelytron mechanism in the last common ancestor of the large Coreini + Gonocerini (part) + Phyllomorphini + Prionotylini clade. This same mechanism also occurred in *Scolopocerus* but was not found in the common ancestor of *Scolopocerus* + *Catorhintha*.

## 4. Discussion

Communication is essential to fitness in many organisms, and the variety of modalities used to communicate is staggering across the animal kingdom. Evolutionary studies of animal communication often focus on signal variation within species and closely related species, but the evolutionary origins and diversification of communication mechanisms—as well as why some lineages evolve a given mechanism but not others—remain puzzling to biologists [88]. Examining stridulatory morphology provides a tractable opportunity to trace the origin and diversification of one kind of communication mechanism. We observed four distinct stridulatory mechanisms within the Coreoidea, with at least five evolutionary gains and no losses of stridulatory mechanisms—suggesting selection to retain stridulatory structures after they evolve.

### 4.1. An updated understanding of the distribution of stridulatory mechanisms in Coreoidea

Our understanding of the presence and diversity of stridulatory mechanisms within the Coreoidea has been limited and hindered by the relatively small taxon sampling of past comparative morphological studies. In turn, this has impeded the ability to investigate the evolution of these mechanisms in a phylogenetic framework and stimulate additional behavioural and bioacoustic/biotremology research in the superfamily. Here, by greatly expanding our taxonomic sampling for investigating the presence or absence of stridulatory mechanisms and placing this in a phylogenetic framework, we provide fertile ground for studies of the selective factors that promote the origin and maintenance of stridulation.

Our broad comparative approach also allowed us to re-evaluate earlier studies on putative stridulatory mechanisms in Coreoidea. By contrast to Miller [46], we did not find evidence of stridulatory mechanisms in species of *Rhyticoris.* Schaefer [40] evaluated the mechanism described by Miller in *Rhyticoris* and other genera, determined that Miller's ‘plectrum’ was an axillary spur on the metathoracic wing and concluded that the axillary spur likely functions as a wing-coupling device rather than as a sound-producing structure. We also do not support Schaefer's [40] conclusion that *Euthochtha galeator* possesses the pronotum–hemelytron mechanism described by Štys [44] after our examination of several specimens of this species did not reveal any stridulatory mechanisms. It may be that Schaefer used misidentified material for this taxon.

We also note that the Cu vein of the metathoracic wing might appear to be a stridulatory structure in some species of Alydidae and Pseudophloeinae. Basally, this vein appears rugose, weakly sclerotized (at most), and without prominent transverse ridges (e.g. figure 6*f*), unlike what is observed in the rhopalids (e.g. figure 6*b* and *d*). Thus, we concluded that species of Alydidae and Pseudophloeinae do not possess a stridulatory mechanism involving the metathoracic wing veins and the thorax and/or abdomen.

### 4.2. Function of stridulatory mechanisms

The presence of structures that resemble a stridulatory mechanism does not confirm vibroacoustic signal production via stridulation. Indeed, Zuk *et al*. [89] elegantly showed how quickly stridulation can be lost in the field cricket, *Teleogryllus oceanicus* (Le Guillou, 1841) (Orthoptera: Gryllidae). Hawaiian populations of *T. oceanicus* have encountered strong natural selection against stridulation due to an introduced parasitoid fly that uses stridulatory signals to locate its host. While these crickets still have structures that resemble a plectrum and stridulitrum, the architecture evolved rapid modifications that rendered them silent [89] (although, some populations have recently evolved a new song via further modifications to the stridulatory structures [90]). Our study focused only on the presence (or absence) of stridulatory structures and their evolution within in the Coreoidea; we do not yet know the extent to which the structures documented correspond to signal production for many species.

Within the Coreoidea, high-frequency stridulatory signals have been confirmed using loss-of-function manipulations for only *J. haematoloma*, with low-frequency signals associated with abdominal movement near the thorax [47]. Based on oscillograms and spectrograms of vibroacoustic signals, *Alydus calcaratus* (Linnaeus, 1758), *Coreus marginatus* (Linnaeus, 1758) and *Enoplops scapha* (Fabricius, 1794) also produce low-frequency signals from tymbal-like organs on abdominal tergites I and II and high-frequency signals from stridulatory mechanisms [17,91,92]. Acoustic signals have also been reported from several other coreoid species that possess stridulatory mechanisms: *Centrocoris spiniger* (Fabricius, 1781), *Centrocoris variegatus* Kolenati, 1845, *Phyllomorpha laciniata* (Villiers, 1789), *Spathocera laticornis* (Schilling, 1829), *Pephricus paradoxus* (Sparrman, 1777) and *Tongorma latreillii* (Guérin-Méneville, 1839) ([39,41–43]; M. Forthman, pers. obs.); given that evidence of acoustic signals does not confirm it is produced from stridulatory structures, these species require further experimental testing (similar to the approach in Zych *et al*. [47]) to determine the structures (i.e. stridulatory or tymbal-like) responsible for the observed sounds.

Stridulatory signals in the Heteroptera have been associated with reproductive behaviours (e.g. Corixidae [93,94], Reduviidae [95] and Miridae [31]) and defensive behaviours (e.g. Reduviidae [95]). However, in the Coreoidea, the social contexts in which stridulatory signals are produced and whether ‘songs’ are sexually dimorphic—particularly since most species examined have a mechanism in both sexes—have not been thoroughly investigated. Zych *et al*. [47] studied stridulation in males and females of *J. haematoloma* and found that individuals would stridulate when they physically encountered a conspecific of either sex, but these signals were not produced when physical contact was made by other arthropods. Schaefer & Pupedis [36] suggested that stridulation in the Alydidae may be associated with pre-mating isolation in aggregations of multiple species, but this has yet to be tested. Shestakov [96] concluded that vibratory signals from *C. marginatus* and *S. laticornis* appear to be used in courtship and aggregation, respectively, but it is unclear whether these originated from the prothorax–hemelytron stridulatory mechanism or some non-stridulatory behaviour. Thus, based on our survey of the literature, almost all of the coreoid taxa we found to possess stridulatory mechanisms require further bioacoustic/biotremology study to confirm both the functionality of these structures and their role in social interactions.

Although our survey of stridulatory mechanisms revealed that they are often present in both sexes, genera within the rhopalid tribe Harmostini exhibited sexual dimorphism with respect to the presence of stridulatory mechanisms. The males of these species possess a well-developed stridulitrum on the metathoracic wing's Cu vein, but the stridulitrum is absent in females. If males do produce stridulatory signals, then they may be involved in pre-mating and mating behaviours. For taxa in which both sexes had stridulatory mechanisms, we were unable to investigate whether there is sexual dimorphism in the fine-scale morphology of the structures due to the limited availability of specimens that could be observed with an SEM. One study did not observe any apparent sexual dimorphism in the Alydidae [36]. However, if sexual differences in signal production are observed, more careful examination of the fine-scale morphology using a larger sampling of each sex may be justified.

### 4.3. Evolution of stridulatory mechanisms

A few phylogenetic comparative studies on the evolution of stridulatory mechanisms have been conducted in other groups of arthropods, including ants (Hymenoptera: Formicidae) [97] and crickets, katydids, and grasshoppers (Orthoptera) [98]. As in the Coreoidea, each of these groups include more than one type of stridulatory mechanism among species, which involves a diversity of body regions or variation in the placement of structures on the same body regions. Similar to our study of the Coreoidea, there is also evidence that some stridulatory mechanisms evolved multiple times within the Formicidae and Orthoptera, while others appear to be lineage-specific and evolved once [97,98]. Stridulatory mechanisms have also been secondarily lost in each of these groups, although in Orthoptera, this has been associated with the loss of wings in some taxa or the evolution of a different mechanism in others [97,98]. By contrast, we did not observe secondary losses of stridulatory mechanisms in the Coreoidea, suggesting continued selection for these structures.

Although our data suggest continued selection to retain stridulatory mechanisms, why stridulatory mechanisms have evolved in five clades within the superfamily while the majority of other coreoid taxa lack such mechanisms remains unknown. In discussing several non-exclusive hypotheses below, we considered the vibratory and acoustics aspects of stridulatory structures. First, stridulatory mechanisms may have evolved due to selection on vibratory signalling repertoires. Several species of the Coreoidea with and without stridulatory mechanisms have been documented to produce low frequency vibrations, such as those from the abdominal tymbal-like organs (e.g. [17,39,41–43,47,91,92])—which is a relatively widespread phenomenon in the Heteroptera [17,91,92]. These low frequency signals are used in, for example, mate location and aggregation of conspecifics in other Heteroptera [17]. Selection pressures may have promoted the evolution of a novel mechanism to produce vibrations with a broader frequency range than those generated by tremulation or tymbal-like organs. Broadening the frequency range could enhance species- and sex-specific signals, with high frequency components possibly involved in species recognition when individuals are in close proximity given the higher attenuation of these signals makes them less relevant for long-distance communication through plant substrate [17,99,100]. Furthermore, in the context of multicomponent (acousto-)vibratory signalling, the evolution of another signalling mode may also be promoted if it expands the potential information content of the emitted signals in a combined display.

A second hypothesis is that stridulation in Coreoidea may have evolved to enhance or modify other means of communication. For example, coreoids—like many other heteropterans—may use chemical signals to facilitate aggregations of individuals, mating, or defence. Stridulation may enhance information conveyed by chemicals, similar to multimodal signalling in some ant species [101]. Additionally, stridulation may also give species the ability to shift between communication modalities (e.g. between visual and vibroacoustic signals) when a given environment impairs the efficacy of one of the signals, as has been suggested in some spiders [102].

Third, stridulatory mechanisms may have evolved in response to predation and parasitic pressures. Aside from invertebrate predators and parasitoids, coreoids are likely predated on by vertebrate species, including birds, mammals and reptiles. Some coreoid species produce sounds that can be detected by humans when disturbed ([36,38,42,43,45]; M. Forthman, pers. obs, 2017–2022) and probably other vertebrate animals, and these acoustic signals likely originate from stridulation. Thus, stridulatory mechanisms may have arisen to produce acoustic warning signals to predators and vibratory signals to warn conspecifics of predatory threats.

Lastly, the evolutionary gain of stridulatory mechanisms in few coreoid clades may simply be because the mutations associated with their development—which would also require the presence of associated stridulatory behaviours—have not arisen in other clades. It will be exciting to examine the behavioural ecology of the coreoid species with and without stridulation in the future to better understand the contexts in which stridulation evolves.

### 4.4. Stridulatory mechanisms as putative synapomorphies and phylogenetic placement of new taxa

Our results suggest the presence of specific stridulatory mechanisms could be useful in diagnosing clades within the Coreoidea. Two of these mechanisms appear to be synapomorphies for taxa within the Rhopalidae: the metawing Cu-thorax/abdomen mechanism for the tribe Harmostini and the metawing Sc+R-abdomen mechanism for the genus *Jadera*. The hemelytron–metafemur mechanism observed in the Alydidae may also diagnose a single clade of all genera possessing it, but this will require further phylogenetic testing when species of *Tollius* and *Euthetus* Dallas, 1852 are available for sampling. The prothorax–hemelytron mechanism may also be used as a diagnostic character within the Coreidae, despite evidence it has evolved independently twice, once in the Coreini + Gonocerini (part) + Phyllomorphini + Prionotylini clade and another in a genus of Hypselonotini (i.e. *Scolopocerus*). However, additional morphological studies of other character systems will be needed to identify additional traits that would diagnose each clade in conjunction with the prothorax–hemelytron mechanism.

The presence of the prothorax–hemelytron mechanism may also be informative in future changes to coreoid classification. The non-monophyly of Gonocerini has been supported in recent phylogenomic studies [51], with species of *Cletoliturus* Brailovsky, 2011, *Cletomorpha* Mayr, 1866 and *Cletus* Stål, 1860 forming a close relationship with the Coreini and Phyllomorphini. As mentioned above, our study recovered this large clade (including Prionotylini), but we also recovered another clade of Gonocerini (including *Gonocerus* and *Plinachtus*) that was more closely related to the Homoeocerini—both of which lack stridulatory mechanisms. Thus, our phylogenetic results, ASE analyses and morphological survey suggest the composition of Gonocerini may need to be revised.

## 5. Conclusion

Our study identified four stridulatory mechanisms within the Coreoidea, which evolved independently at least five times. While stridulatory signal production is likely involved in intraspecific social interactions (e.g. aggregation and mating), this has yet to be experimentally investigated for the vast majority of coreoid species. In fact, while vibroacoustic signals have been documented for several coreoid species, the origins of these remain unknown for most (i.e. stridulatory versus non-stridulatory). One stridulatory mechanism is described in the superfamily for the first time (metawing Cu-thorax/abdomen mechanism), and sexual dimorphism in the presence of this mechanism suggests the use of stridulation may also differ for taxa in the Harmostini relative to other coreoid species. Given these gaps in knowledge, further behavioural, bioacoustic/biotremology and natural history studies in taxa with and without stridulatory mechanisms will expand our knowledge on the functionality and communicative purpose of stridulatory mechanisms in Coreoidea. Addressing these gaps in knowledge will also facilitate hypothesis testing regarding the evolutionary origins and maintenance of stridulatory communication and whether there is a reduction in other forms of communication when stridulation is present. We hope that this study provides a foundation from which future work can link form to function, documenting signals, their variability within and across species, and ultimately the factors that select for the gains and modifications of stridulation in the superfamily Coreoidea.

## Data Availability

Sequence read files of newly generated data are available on NCBI's Sequence Read Archive under BioProject PRJNA878845. Alignments, gene trees and concatenation and species trees are available from FigShare under the project titled ‘Evolution of stridulatory mechanisms: vibroacoustic communication may be common in leaf-footed bugs and allies (Heteroptera: Coreoidea)’ (https://figshare.com/projects/Evolution_of_stridulatory_mechanisms_vibroacoustic_communication_may_be_common_in_leaf-footed_bugs_and_allies_Heteroptera_Coreoidea_/148711) [103]. Supplementary material is available online [104].
